# Supplementary material for: The Effects of Dairy and Plant-Based Liquid Components on Lutein Liberation in Spinach Smoothies
Source: Nutrients. 2023 Feb 2;15(3):779. doi: 10.3390/nu15030779 (PMC9920929; doi:10.3390/nu15030779)
Supplement: Supplementary file 1 [file nutrients-15-00779-s001.zip › nutrients-2142420-supplementary.docx]

Supplementary Materials of

The effects of dairy and plant-based liquid components on lutein liberation in spinach smoothies

Jan Neelissen^1^, Per Leanderson^2^, Lena Jonasson^1^ and Rosanna W.S. Chung^1*^

Supplementary Table 1: Detailed information of the dairy products used

| Liquid Components | Low fat  yoghurt | Mild  yoghurt | Greek  yoghurt | Low fat cow’s milk | Medium fat cow’s milk | High fat cow’s milk | Whipping cream |
| --- | --- | --- | --- | --- | --- | --- | --- |
| Product names on the package (Swedish) | Ko mild lättyoghurt naturell 0.5% | Ko yoghurt naturell 3% | Ko mild  yoghurt Grekisk 6% | Ko färsk lättmjölk 0.5% EKO | Ko färsk mellanmjölk 1.5% EKO | Ko Färsk standardmj-ölk 3.0% EKO | Vispgrädde 36% |
| Product names (English) | Cow’s low fat yoghurt 0.5% | Cow’s medium fat yoghurt 3% | Cow’s Greek  yoghurt 6% | Organic cow fresh low fat milk 0.5% | Organic cow fresh medium fat milk 1.5% | Organic cow’s Fresh high fat milk 3% | Whipping cream 36% |
| Brands | Arla^c^ | Arla^c^ | Arla^c^ | Arla^c^ | Arla^c^ | Arla^c^ | ICA^d^ |
| Fat content (g/100g) | 0.5 | 3 | 6 | 0.5 | 1.5 | 3 | 36 ^a^ |
| Saturated fat | 0.3 | 1.9 | 3.8 | 0.3 | 1.0 | 1.9 | 23 ^a^ |
| Carbohydrate content (g/100g) | 4.0 | 3.6 | 3.6 | 4.9 | 4.9 | 4.8 | 3.2 ^a^ |
| Sugars | 4.0 | 3.6 | 3.6 | 4.9 | 4.9 | 4.8 | 3.2 ^a^ |
| Protein content (g/100g) | 4.1 | 3.4 | 3.3 | 3.5 | 3.5 | 3.4 | 2.3 ^a^ |
| Salt (g/100g) | 0.1 | 0.1 | 0.1 | 0.1 | 0.1 | 0.1 | 0.1 ^a^ |
| Vitamin D1 (µg/100g) | 1.0 | 1.0 |  | 1.0 | 1.0 |  |  |
| Vitamin A (µg/100g) |  |  |  |  |  |  | 330 ^b^ |
| Vitamin B12 (µg/100g) |  |  |  | 0.6 | 0.6 | 0.6 | 0.4 ^b^ |
| Riboflavin (mg/100g) |  |  |  | 0.15 | 0.15 | 0.15 |  |
| Pantothenic acid (mg/100g) |  |  |  | 0.5 | 0.5 | 0.5 |  |
| Folic acid (µg/100g) |  |  |  | 15 | 15 | 15 |  |
| Calcium (mg/100g) | 130 | 120 | 120 | 120 | 120 | 120 |  |
| Potassium (mg/100g) |  |  |  | 160 | 160 | 160 |  |
| Chloride (mg/100g) |  |  |  | 95 | 95 | 95 |  |
| Phosphorus (mg/100g) |  |  |  | 105 | 105 | 105 |  |
| Molybdenum (µg/100g) |  |  |  | 4.7 | 4.7 | 4.7 |  |
| Iodine (µg/100g) |  |  |  | 12 | 12 | 12 |  |

All information on nutrition content was obtained from the product packaging which was written in Swedish. All Swedish-English translations were performed by the authors. ^a^ Nutrient concentration listed in g/100mL. ^b^ Nutrient concentration listed in µg/100mL. ^c^ Abbreviation for Arla Foods AB. ^d^ Abbreviation for ICA Gruppen AB.

Supplementary Table 2: Ingredient list of the dairy products used

| Liquid Components | Ingredient list (in order of descending quantity) |
| --- | --- |
| Low fat yoghurt | Highly pasteurized milk, milk protein, mild yoghurt bacterial culture, vitamin D |
| Mild yoghurt | Highly pasteurized milk, yoghurt bacterial culture, vitamin D |
| Greek yoghurt | Highly pasteurized milk and cream, Greek mild yoghurt bacterial culture |
| Low fat cow’s milk | Highly pasteurized milk, vitamin D |
| Medium fat cow’s milk | Highly pasteurized milk, vitamin D |
| High fat cow’s milk | Highly pasteurized milk |
| Whipping cream | Highly pasteurized cream, stabilizer (carrageenan) |

All information on nutrition content was obtained from the product packaging which was written in Swedish. All Swedish-English translations were performed by the authors.

Supplementary Table 3: Detailed information on the plant-based products used

| Liquid Components | Pure soymilk | Soymilk with additives | Pure oat milk | Oat milk with  additives | Almond milk with additives | Pure  coconut milk | Coconut milk with additives |
| --- | --- | --- | --- | --- | --- | --- | --- |
| Product names on the package (Swedish or English) | Ekologisk sojadryck | Sojadryck utan socker | Havre-dryck Ekologisk | Havre-dryck | Rostad mandeld-ryck utan socker | Coconut milk original | Kokos-dryck utan socker |
| Translated product names (English) | Organic Soymilk | Soymilk without sugars | Organic oat drink | Oat milk | Roasted Almond milk without sugars | Coconut milk original | Coconut without sugars |
| Brands | Alpro^b^ | Alpro^b^ | Oatly^c^ | Oatly^c^ | Alpro^b^ | Santa Maria^d^ | Alpro^b^ |
| Fat content (g/100mL) | 1.8 | 1.8 | 0.5 | 1.5 | 1.1 | 18 ^a^ | 1.2 |
| Saturated fat | 0.3 | 0.3 | 0.1 | 0.2 | 0.1 | 16.4 ^a^ | 1.1 |
| Monounsaturated fat | 0.3 | 0.4 |  |  | 0.7 |  | 0.1 |
| Polyunsaturated fat | 1.1 | 1.1 |  |  | 0.3 |  | 0 |
| Carbohydrate content (g/100mL) | 2.3 | 0 | 6.7 | 6.7 | 0 | 2.7 ^a^ | 0 |
| Sugars | 2.3 | 0 | 4.1 | 4.1 | 0 | 2.2 ^a^ | 0 |
| Protein content (g/100mL) | 3.0 | 3.3 | 1.0 | 1.0 | 0.4 | 1.9 ^a^ | 0.1 |
| Fiber (g/100mL) | 0.5 | 0.6 | 0.8 | 0.8 | 0.3 |  | 0 |
| Salt (g/100mL) | 0.14 | 0.09 | 0.10 | 0.10 | 0.14 | 0.02 ^a^ | 0.07 |
| Vitamin D2 (µg/100mL) |  | 0.75 | 1.10 | 1.10 | 0.75 |  | 0.75 |
| Vitamin E (mg/100mL) |  |  |  |  | 1.80 |  |  |
| Vitamin B12 (µg/100mL) |  | 0.38 |  | 0.38 | 0.38 |  | 0.38 |
| Riboflavin (mg/100mL) |  | 0.21 |  | 0.21 | 0.21 |  |  |
| Calcium (mg/100mL) |  | 120 |  | 120 | 120 |  | 120 |

All information on nutrition content was obtained from the product packaging which was written in Swedish except for pure coconut milk. All Swedish-English translations were performed by the authors. ^a^ Nutrient concentration listed in g/100g. ^b^ Alpro is a part of Danone AB. ^c^ Abbreviation for Oatly Group AB. ^d^ Abbreviation for Santa Maria AB.

Supplementary Table 4: Ingredient list of the plant-based products used

| Liquid Components | Ingredient list (in order of descending quantity) |
| --- | --- |
| Pure soymilk | Soya base (water, hulled soya beans (8%)), raw cane sugar, sea salt. |
| Soymilk with additives | Soya base (water, hulled soya beans (8.7%)), acidity regulators (Potassium phosphates), calcium carbonate, flavorings, sea salt, stabilizer (gellan gum), vitamins (B2, B12, D2). |
| Pure oat milk | Oat base (water, oats (10%)), sea salt, vitamins D2. |
| Oat milk with additives | Oat base (water, oats 10%), rapeseed oil, calcium carbonate, calcium phosphates, salt, vitamins (D2, riboflavin, B12), potassium iodide. |
| Almond milk with additives | Water, almond (2.3%), tri-calcium phosphate, sea salt, stabilizers (locust bean gum, gellan gum), emulsifier (lecithins (sunflower)), vitamins (B2, B12, E, D2). |
| Pure coconut milk | Pressed coconut (18% fat), water. |
| Coconut milk with additives | Water, coconut milk (7%) (coconut cream, water), coconut water (2,6%), tri-calcium phosphate, natural coconut flavor, stabilizers (guar gum, xanthan gum, gellan gum), Sea salt, vitamins (B12, D2). |

All information on nutrition content was obtained from the product packaging which was written in Swedish. All Swedish-English translations were performed by the authors.


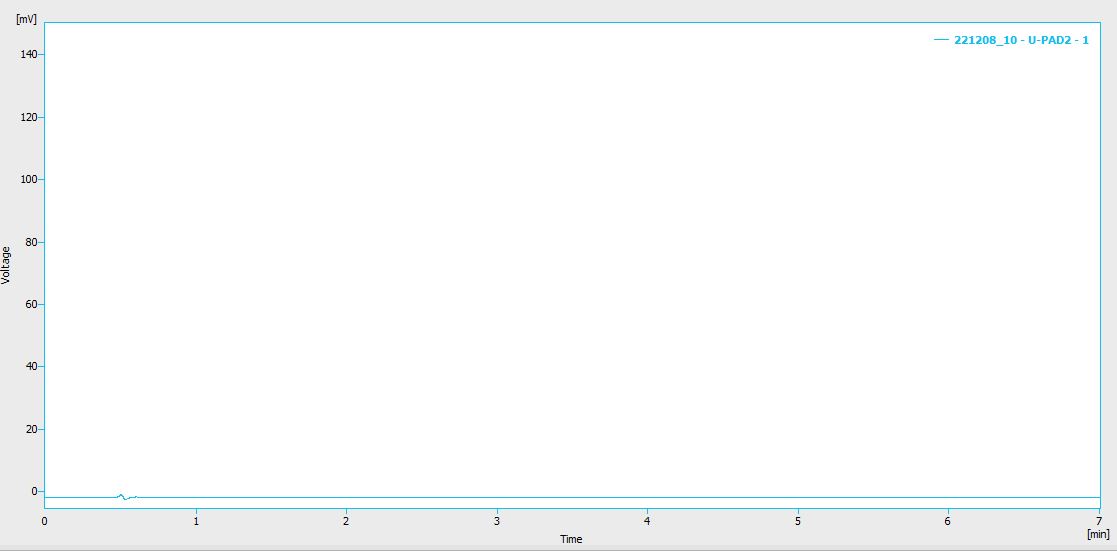


Supplementary Figure 1: Representative chromatogram of carotenoid extracts from digestate of smoothie made of milk alone


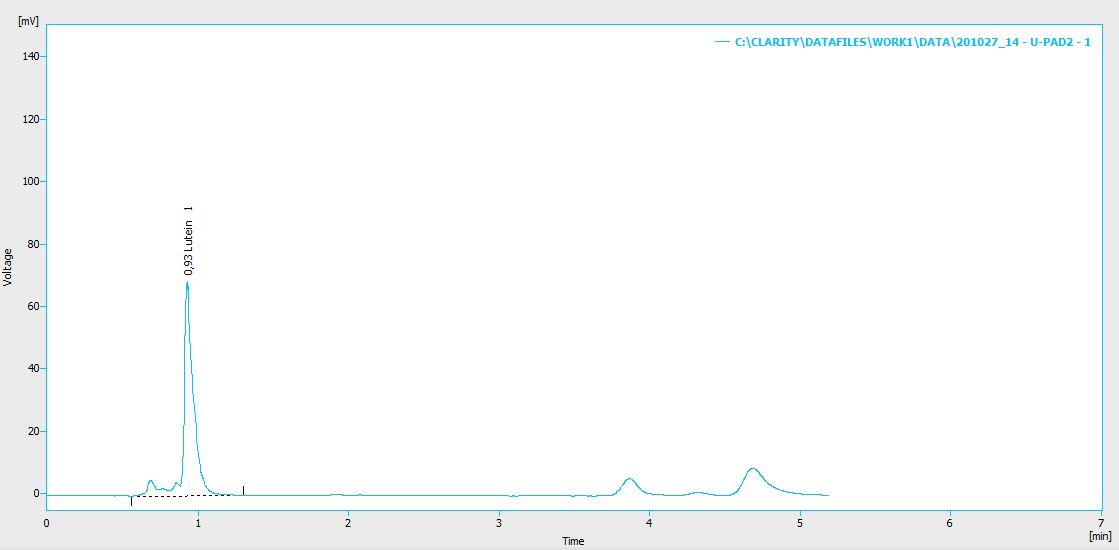


Supplementary Figure 2: Representative chromatogram of smoothie made of high fat cow’s milk and spinach.


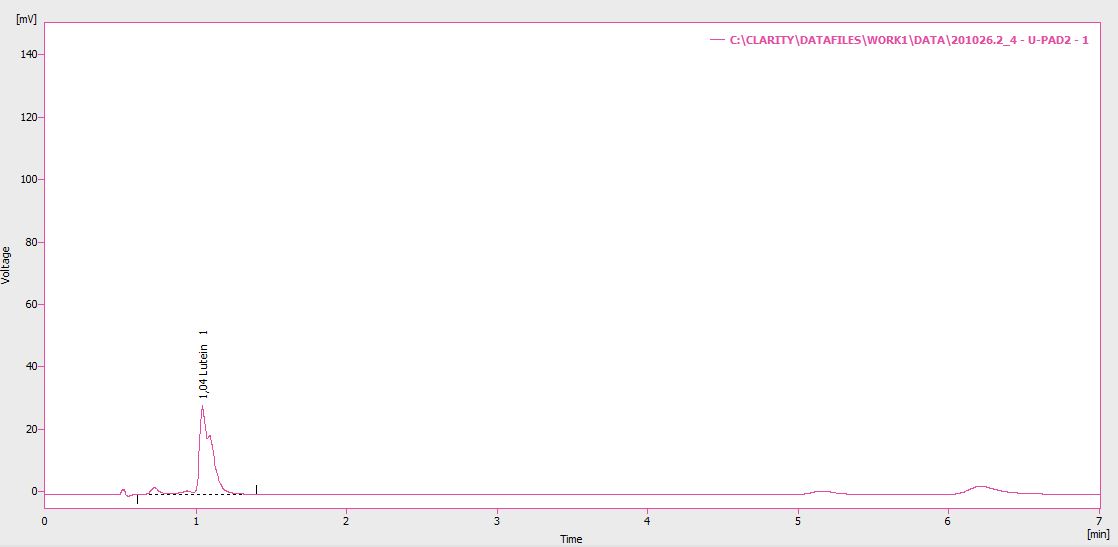


Supplementary Figure 3: Representative chromatogram of smoothie made of low fat yoghurt and spinach.


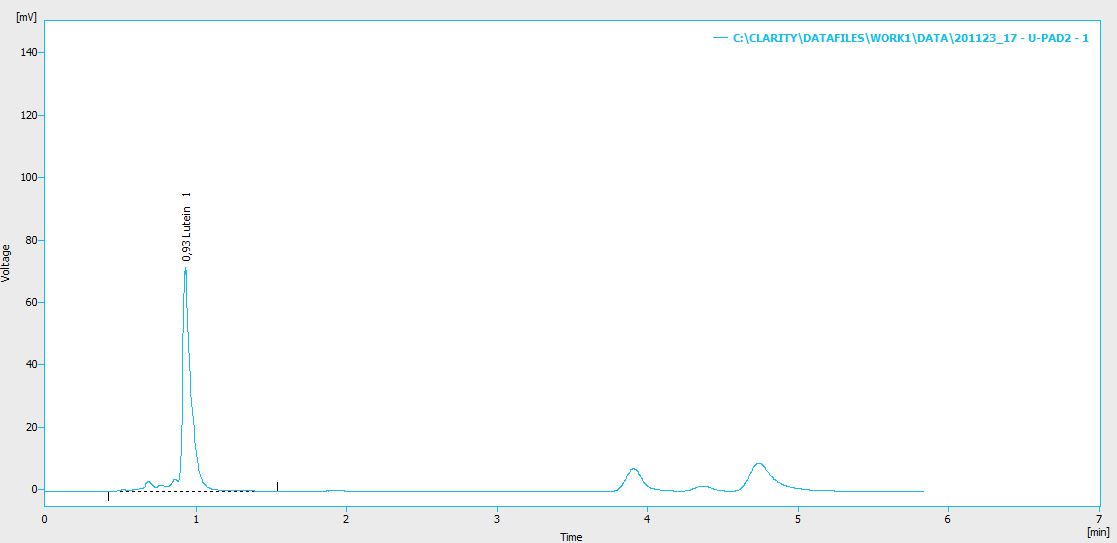


Supplementary Figure 4: Representative chromatogram of smoothie made of pure coconut milk and spinach.

………………………………….
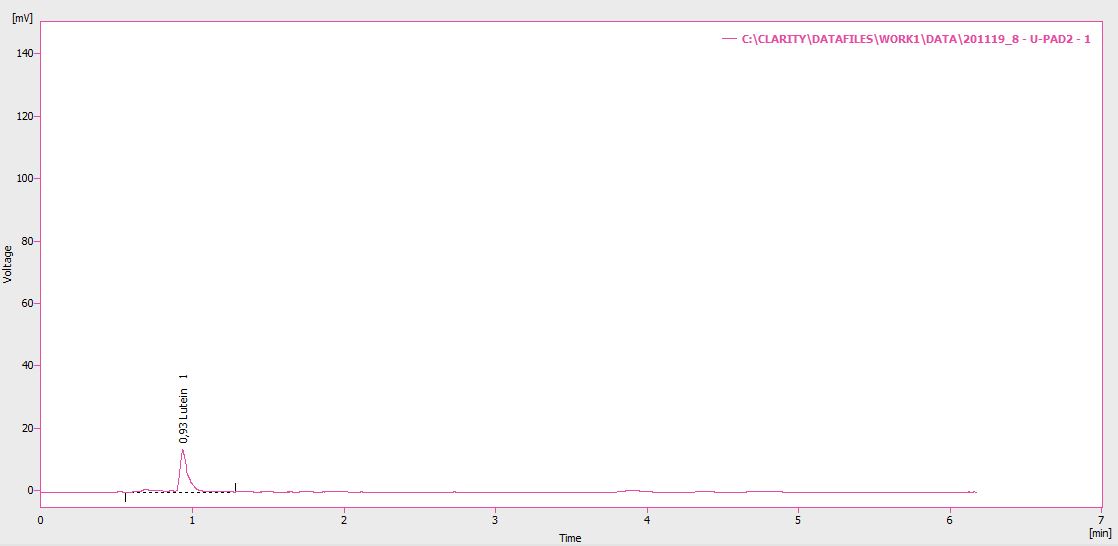


Supplementary Figure 5: Representative chromatogram of smoothie made of pure soymilk and spinach.

**Supplementary Table 5.** Multiple comparisons between liquid types in terms of improvement in lutein liberation per gram of fat before and after adjustment for protein.

|  | **Unadjusted model** | | | | | **Adjusted model** | | | | |
| --- | --- | --- | --- | --- | --- | --- | --- | --- | --- | --- |
|  | **High fat cow´s milk** | **Coconut milk with additives** | **Pure coconut milk** | **Soymilk with additives** | **Pure soymilk** | **High fat cow´s milk** | **Coconut milk with additives** | **Pure coconut milk** | **Soymilk with additives** | **Pure soymilk** |
| Medium fat cow’s  milk | ns | ns | ns | <0.001 | <0.001 | ns | ns | 0.002 | <0.001 | <0.001 |
| High fat cow´s milk |  | ns | ns | <0.001 | <0.001 |  | ns | ns | <0.001 | <0.001 |
| Coconut milk with additives |  |  | ns | <0.001 | <0.001 |  |  | ns | ns | 0.049 |
| Pure coconut milk |  |  |  | <0.001 | <0.001 |  |  |  | <0.001 | <0.001 |
| Soymilk with  additives |  |  |  |  | ns |  |  |  |  | ns |

The unadjusted model was generated by ANOVA multiple comparisons with Bonferroni correction; The adjusted model was generated by ANCOVA multiple comparisons with Bonferroni correction using the effect of protein as a co-variant. Each cell indicates p-value between two types of liquids. “ns” represents not significant.

**Supplementary Table** 6. Multiple comparisons between liquid types in terms of improvement in lutein liberation per gram of protein before and after adjustment for fat

|  | **Unadjusted model** | | | | | **Adjusted model** | | | | |
| --- | --- | --- | --- | --- | --- | --- | --- | --- | --- | --- |
|  | **High fat cow´s milk** | **Coconut milk with additives** | **Pure coconut milk** | **Soymilk with additives** | **Pure soymilk** | **High fat cow´s milk** | **Coconut milk with additives** | **Pure coconut milk** | **Soymilk with additives** | **Pure soymilk** |
| Medium fat cow’s  milk | ns | <0.001 | ns | ns | ns | ns | <0.001 | 0.002 | ns | ns |
| High fat cow´s milk |  | <0.001 | ns | ns | ns |  | <0.001 | 0.010 | ns | ns |
| Coconut milk with additives |  |  | <0.001 | <0.001 | <0.001 |  |  | <0.001 | ns | ns |
| Pure coconut milk |  |  |  | 0.011 | 0.005 |  |  |  | ns | ns |
| Soymilk with  additives |  |  |  |  | ns |  |  |  |  | ns |

The unadjusted model was generated by ANOVA multiple comparisons with Bonferroni correction; The adjusted model was generated by ANCOVA multiple comparison with Bonferroni corrections using the effect of fat as a co-variant. Each cell indicates p-value between two types of liquids. “ns” represents not significant.
